# Kaempferol Alleviates Oxidative Stress and Apoptosis Through Mitochondria-dependent Pathway During Lung Ischemia-Reperfusion Injury

**DOI:** 10.3389/fphar.2021.624402

**Published:** 2021-03-04

**Authors:** Chunli Yang, Wenkai Yang, Zhaohui He, Jinghua Guo, Xiaogang Yang, Rongsheng Wang, Hongbo Li

**Affiliations:** ^1^ Department of Intensive Care, Jiangxi Provincial People’s Hospital Affiliated to Nanchang University, Nanchang, China; ^2^ Department of Cardiovascular Surgery, Central People's Hospital of Zhanjiang, Zhanjiang, China; ^3^ Department of Orthopedics, Jiangxi Provincial People’s Hospital Affiliated to Nanchang University, Nanchang, China

**Keywords:** kaempferol, lung ischemia-reperfusion injury, mitochondria, apoptosis, oxidative stress, SIRT1, PGC-1α

## Abstract

In previous study, we reported that kaempferol ameliorates significantly lung ischemia-reperfusion injury (LIRI), and may be achieved by targeting the SIRT 1 pathway. This study further explored the anti-LIRI mechanism of kaempferol. *In vitro*, the rat alveolar epithelial cells L2 was cultured and subjected to anoxia/reoxygenation (A/R) insult. *In vivo*, SD rats were operated to establish LIRI model. The related indicators of oxidative stress and apoptosis in L2 cells and rats lung tissues were detected. Results showed that kaempferol pre-treatment significantly increased the cell viability, improved mitochondrial membrane potential, inhibited the opening of mitochondrial permeability transition pores, reduced the levels of oxidative stress and apoptosis, increased the expressions of Bcl-2 and mitochondrial cytochrome c, and decreased the expressions of Bax and cytoplasmic cytochrome c in L2 cells after A/R insult. *In vivo*, kaempferol improved the pathological injury, inhibited the levels of oxidative stress and apoptosis, increased the expressions of Bcl-2 and mitochondrial cytochrome c, and decreased the expressions of Bax and cytoplasmic cytochrome c in rats lung tissues after I/R. However, the aforementioned effects of kaempferol were significantly attenuated by the SIRT 1 inhibitor EX527 or the PGC-1α inhibitor SR-18292. What’s more, SR-18292 has not reversed the effect of kaempferol on increasing the protein activity of SIRT 1. Above results suggest that kaempferol ameliorates LIRI by improving mitochondrial function, reducing oxidative stress and inhibiting cell apoptosis. Its molecular mechanism of action includes the SIRT 1/PGC-1α/mitochondria signaling pathway.

## Introduction

Lung ischemia-reperfusion injury (LIRI) is a common acute lung injury in clinic, which can lead to inflammation, oxidative stress and cell death in lung, threatening the lives of patients (Fernandez et al., 2013). The development of new therapies or drugs to prevent and treat LIRI is an urgent matter (Bonservizi et al., 2014). In our previous study, we found that kaempferol, a natural flavonoid, has a significant effect on anti-LIRI in rats, and its mechanism may be achieved through targeting activation of sirtuin (SIRT) 1 protein (Yang et al., 2020). However, its specific mechanism still needs to be further improved.

The working environment of the lung determines that the lung cells are exposed to high oxygen and high blood supply environment for the long time, and are extremely vulnerable to oxidative stress (Park et al., 2010; Bhatia, 2017). Reactive oxygen species (ROS) are the key factors that inducing oxidative stress. Under normal circumstances, they participate in the regulation of specific signal pathways to maintain the normal functions of cells (Finkel and Holbrook, 2000). Endogenous ROS are mainly produced by mitochondria. When ROS are produced in large quantities and cannot be eliminated in time, they will cause mitochondrial dysfunction, which plays a crucial role in bioenergetics metabolism and non-energetics pathogenesis in many lung diseases (Schumacker et al., 2014). During the ischemia-reperfusion (I/R) process, mitochondria produce excessive ROS, leading to DNA damage, mitochondrial lipid peroxidation, Ca^2+^ homeostasis destruction and mitochondrial membrane depolarization, followed by the release of cytochrome c into the cytoplasm to induce apoptosis (Mattson et al., 2000; Murphy and Steenbergen, 2008; Lemasters et al., 2009; Wu and Bratton, 2013). Therefore, mitochondrial function is a key determinant of cell survival during ischemic injury (Ham and Raju, 2017). The expression and/or activity of SIRT 1 is a known important way to promote mitochondrial biogenesis (Valero, 2014). We speculate that kaempferol targets SIRT 1 through mitochondrial-dependent pathways to exert anti-oxidative stress and anti-apoptosis effects to improve LIRI. The aim of this study is to explore the relationship between kaempferol’s anti-LIRI effect and mitochondrial function *in vitro* and *in vivo*, and to further improve the anti-LIRI mechanism of kaempferol.

## Materials and Methods

### Reagents

Kaempferol (purity >98%) was obtained from the National Institute for the Control of Pharmaceutical and Biological Products (Beijing, China), and dissolved in dimethylsulfoxide (30 mg/ml), with a final concentration in phosphate buffer solution (PBS). SIRT1 inhibitor EX527 and peroxisome proliferator activated receptor gamma coactivator-1α (PGC-1α) inhibitor SR-18292 were bought from the Selleckchem (Houston, TX, United States), and both dissolved in dimethylsulfoxide (both were 30 mg/ml), with a final concentration in PBS. The antibodies of B-cell lymphoma 2 (Bcl-2), Bcl-2-associated X (Bax), cytochrome c (Cyt c) and cytochrome c oxidase subunit IV (COX IV) were all obtained commercially from Abcam (Cambridge, MA, United States). The detection kits of reactive oxygen species (ROS), malondialdehyde (MDA), total superoxide dismutase (SOD), glutathione (GSH) and glutathione peroxidase (GSH-Px) were purchased from Jiancheng (Nanjing, JS, CHN). Annexin V-FITC apoptosis detection kit obtained from eBioscience (San Diego, CA, United States).

### Cell Culture

The L2 cell line, rat alveolar epithelial cells, was purchased from Procell (Wuhan, HB, CHN) and grown in Ham’s F-12k with 10% calf bovine serum and 1% penicillin-streptomycin at 37°C with 5% CO_2_ (v/v). Medium was replaced two to three days and the cells were passaged when the cell adherence area reached 80% of the culture dish.

### Experimental Groups and Treatments

The L2 cells were randomly divided into the following groups. 1) The control group (Control), in which cells were incubated with vehicle for 52 h 2) Anoxia/reoxygenation (A/R) group (A/R), in which the cells were incubated with vehicle for 24 h, and then exposed to 24 h of anoxia (1% O_2_-5% CO_2_-94% N_2_) followed by 4 h of 5% CO_2_-95% air (reoxygenation) as reported (Wu et al., 2013). 3) Kaempferol pre-treatment group (A/R + Kae), in which the cells were incubated with 10, 20 and 40 μm kaempferol for 24 h before initiation of A/R as the A/R group. 4) Kaempferol and EX527 combination pre-treatment group (A/R + Kae + EX527), in which the cells were co-incubated with 40 μm kaempferol and 10 μm EX527 for 24 h before initiation of the A/R as the A/R group. 5) Kaempferol and SR-18292 combination pre-treatment group (A/R + Kae + SR-18292), in which the cells were co-incubated with 40 μm kaempferol and 10 μm SR-18292 for 24 h before initiation of the A/R as the A/R group. The doses of EX527 and SR-18292 performed in present experiments were determined during the preliminary experiments.

### MTT Assay

3-(4,5-dimethylthiazol-2-yl)-5-(3-carboxymethoxyphenyl)-2-(4-sulfophenyl)-2H-tetrazolium (MTT; Promega, WI, United States) assay was used to evaluate the effects of kaempferol and EX527 or SR-18292 on L2 cells viability followed the methods of Chunli Yang et al. (Cai et al., 2020). Briefly, the cells were plated into 96-well plates with a density of 1 × 10^5^ cells/well. After 24 h of culture, adherent cells were treated with kaempferol and EX527 or SR-18292 for 24 h before initiation of the A/R. After A/R, cells were incubated with 20 μl MTT (5 mg/ml) in 100 μl cell culture medium for 4 h at 37°C. After 4 h, the absorbance of each well was measured at a wavelength of 490 nm.

### Assessment of Oxidative Stress

ROS assay kit (Jiancheng Bioengineering Institute, CHN) was used to detect the level of ROS in L2 cells followed the methods of Chunli Yang et al. (Cai et al., 2020). Briefly, tryptase digestion was used to obtain cell suspension and 2′,7′-dichlorofluorescein was used as probe. Flow cytometry was performed to detect the levels of ROS in cells and performed according to the manufacturer’s instruction. The levels of MDA, SOD, GSH and GSH-PX in L2 cells were detected by corresponding kits (Jiancheng, CHN) in accordance with the instructions.

### Assessment of Apoptosis

As previous (Cai et al., 2020), trypsin-EDTA was used to obtain single-cell suspension after incubating 10 min, then washed the cells with chilled D-Hanks (pH = 7.2–7.4) after centrifugation, and incubated in Annexin-V binding buffer for 15 min at room temperature, which containing Annexin-V-APC. Flow cytometry (Becton Dickinsonm, United States) was used to quantify the fluorescence of Annexin-V-APC (Millipore, United States) with a minimum of 10,000 cells counted for each group.

### Assessment of Mitochondrial Membrane Potential

Mitochondrial membrane potential (Δψm) was detected by fluorescent dye tetrechloro-tetraethylbenzimidazol carbocyanine iodide (JC-1) (Invitrogen, United States). Briefly, L2 cells were collected and resuspended, then incubated with JC-1 (200 μm) for 20 min at 37°C followed by washing twice with PBS for each 30 s. Flow Cytometer (Becton Dickinsonm, United States) was used to measure the fluorescence at excitation and emission wavelengths (ex/em) of 530 nm and 580 nm (“red”) first, and then at ex/em of 485 nm/530 nm (“green”), respectively.

### Assessment of the Opening of Mitochondrial Permeability Transition Pores

Mitochondrial/cytosolic fractionation kit (QIAGEN, Germany) was used to isolated mitochondria of L2 cells. The precipitated mitochondria were resuspended with swelling buffer at a final concentration of 0.25 mg/ml. A Ca^2+^-induced mitochondria swelling assay was then performed to detect the opening of mPTP. The absorbance at 520 nm was continuously measured to reflect the degree of mPTP opening. This decrease in optical density was measured by the change in absorption at 520 nm, the changes in absorbance at 520 nm/min (ΔODmin-1) were used to express the extent of mPTP opening.

### Animals

Adult male Sprague Dawley (SD) rats (250–300 g), obtaining from the Nanchang University Laboratory Animal Center, were allowed to access food and water ad libitum and maintained under a 12 h dark/light cycle at 22–25°C. Experiments were carried out according to the Guide for the Care and Use of Laboratory Animals published by the United States National Institutes of Health (NIH Publication No. 85-23, revised in 1996), and approved by the Ethics Committee of Nanchang University (No. 2019-0311).

### Experimental Groups and Treatments

The rats were randomly divided into five groups with six rats in each as follows. 1) The control group (Control), as previous (Yang et al., 2020), the rats were intraperitoneally injected with 2 ml vehicle for 7 days. One hour after the last administration, rats were anesthetized with pentobarbital sodium (50 mg/kg) intraperitoneally, performed thoracotomy, exposed the trachea and then closed the chest immediately. 2) I/R group, the rats were intraperitoneally injected with 2 ml vehicle for 7 days, then operated 1 h after the last administration as previous reported (Yang et al., 2020). Briefly, after the rats were anesthetized with pentobarbital sodium, the trachea was surgically exposed and mechanical ventilation was performed with 40% O_2_. Open the chest at the left 3–5 intercostal space, free the left hilar, and arterial clip to block the left hilum for 90 min, then restore blood flow and reperfusion for 4 h. Anesthesia was maintained with inhaled halothane. 3) Kaempferol pre-treatment group (I/R + Kae), as previous report (Yang et al., 2020), the rats were intraperitoneally injected with 50 mg/kg kaempferol for 7 days before I/R operation. Surgery was performed as I/R group immediately 1 h after the last administration. 4) Kaempferol and EX527 combination pre-treatment group (I/R + Kae + EX527), the rats were intraperitoneally injected with EX 527 (5 mg/kg, every 2 days, total 7 days) before I/R operation on the basis of I/R + Kae group as previous report (Yang et al., 2020). 5) Kaempferol and SR-18292 combination pre-treatment group (I/R + Kae + SR-18292), the rats were intraperitoneally injected with 30 mg/kg SR-18292 for consecutive 7 days before I/R operation on the basis of I/R + Kae group. The rats in each group were sacrificed after re-perfusion by removing the ventilator. The left lungs were removed out from the thoracic cavity. One part of left lung was weighed, dried at 60°C for 48 h and weighed again to calculate the wet/dry (W/D) ratio. The remaining left lung was frozen directly at −80°C or fixed with 4% paraformaldehyde.

### Assessment of Lung Injury

After being fixed overnight, the left lung tissue was embedded in paraffin and stained with hematoxylin and eosin (HE). Two pathologists blindly scored the lung injury according to the following scoring system (Prakash et al., 2015). It includes the following three items. 1) Aggregation or infiltration of inflammatory cells in vessel wall or air space, and scoring as follows: 1 = only wall and air space, 2 = few cells infiltration in air space, 3 = intermediate, 4 = severe aggregation of inflammatory cells in air space. 2) Hyaline membrane formation and interstitial congestion, and scoring as follows: 1 = normal, 2 = moderate (>25% of lung section), 3 = intermediate (25–50% of lung section), 4 = severe (>50% of lung section). 3) Hemorrhage and scoring as follows: 0 = absent, 1 = present. The total score of each item was the final score.

### Assessment of Oxidative Stress

The left lung tissue was fully homogenized and centrifuged to obtain the supernatant. The MDA, SOD, GSH and GSH-PX in the supernatant were detected by the corresponding detection kits (Jiancheng, CHN). The detection process was strictly implemented in accordance with the instructions provided by the manufacturer.

### TUNEL Staining

Lung apoptosis was examined by the terminal deoxynucleotidyl transferase-mediated deoxyuridine triphosphate-biotin nick end labeling (TUNEL) followed the methods of Chunli Yang et al. (Cai et al., 2020). Briefly, after deparaffinization and rehydration, paraffin sections were incubated with 10 mm proteinase k for 15 min, and then transferred to the TUNEL reaction mixture for 60 min at 37°C in the dark. Finally, incubate with Converter-POD for 30 min. The nucleus of an apoptotic cell will be stained brown, while the nucleus of a normal cell will be blue. The ratio of the number of apoptotic cells to the total number of cells was the apoptosis index. Each slice randomly selected eight areas for statistics by one co-author blindly, and took the average value.

### Western Blotting

As previous (Yang et al., 2020), the total protein, mitochondrial protein and cytoplasmic protein in lung tissue or L2 cells were extracted and separated by sodium dodecyl sulfate - polyacrylamide gel electrophoresis and electrotransferred onto polyvinylidene difluoride membranes that was probed with primary antibodies (1:500) diluted in 5% nonfat milk and incubated at 4°C overnight. The membranes were incubated with the horseradish peroxidase-conjugated secondary antibodies after washing with Tris-buffered saline and Tween 20 (TBS-T). Enhanced chemiluminescence was performed to evaluate the immune complexes and ImageJ v2.1.4.7 software (National Institutes of Health, Bethesda, MD, United States) was used to analyze the band intensity quantitatively.

### SIRT 1 Activity Assay

The total protein in lung tissue or L2 cells were extracted and detected by a SIRT1 activity assay kit (Abcam, United Kingdom) based on instructions of the manufacturer as previous reported (Yang et al., 2020). Measurements of fluorescence intensity was carried out at 340 nm excitation and 460 nm emission using a microtiter plate fluorometer.

### Statistical Analysis

Data were presented as means ± standard error and analyzed by SPSS version 20.0 (IBM Corp., Armonk, NY, United States). The assumption of normality was tested with the Shapiro—Wilk test for normality prior to additional analysis. Normally distributed data were analyzed by Student’s unpaired, two - tailed *t*-test, or one-or two-way ANOVA followed by Dunnett’s or Tukey’s post-hoc test. Data not normally distributed were analyzed using the Mann - Whitney *U*-test or Kruskal -Wallis one -way ANOVA. *p* < 0.05 was set as the level of significance for all comparisons.

## Results

### Kaempferol Improves the Viability of Alveolar Epithelial Cells After A/R

**FIGURE 1:**
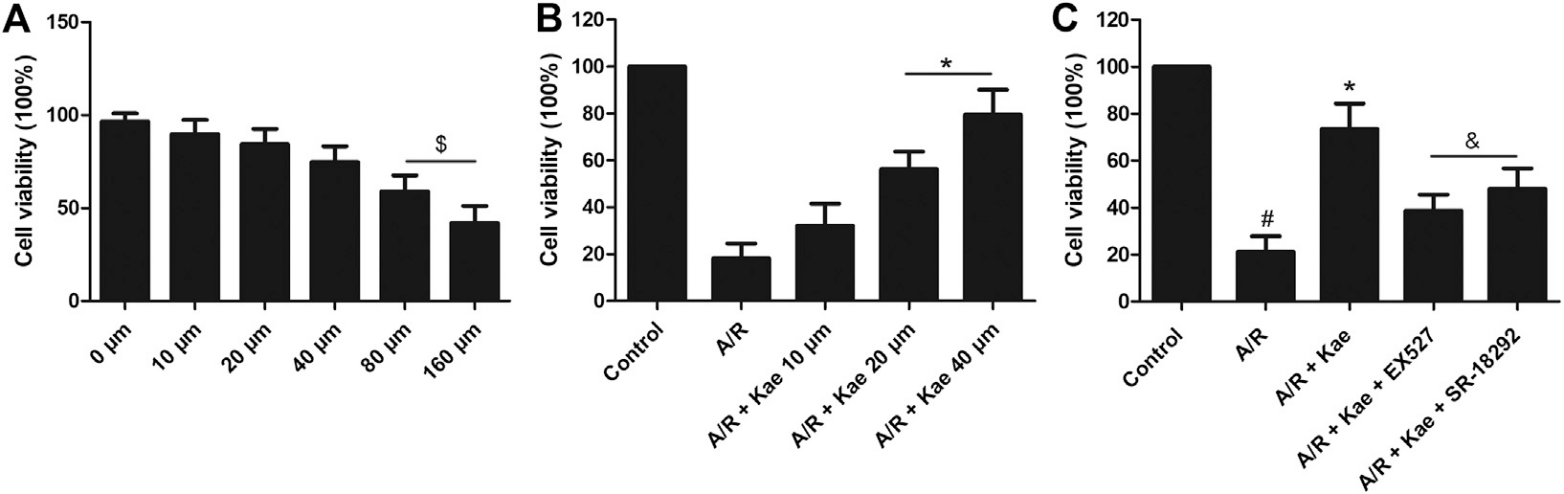
Effects of kaempferol pre-treatment on L2 cells viability. MTT was performed to detect L2 cells viability after pre-treating with kaempferol. **(A)** Effects of kaempferol with different concentrations intervention for 24 h on the viability of normal L2 cells. **(B)** Effects of kaempferol with different concentrations pre-treatment on the viability of L2 cells after A/R insult. **(C)** Effects of kaempferol and EX527 or SR-18292 pre-treatment on the viability of L2 cells after A/R insult. Data are presented as the means ± standard error of the mean for six independent experiments. ^$^ *p*＜0.05 vs. 0 μm, ^#^ *p*＜0.05 vs. the control group, *p＜0.05 vs. the A/R group, ^&^ *p*＜0.05 vs. the A/R + Kae group.

We first observed the effects of different doses of kaempferol on L2 normal cells. As shown in Figure 1A, as the concentration of kaempferol increased, cell viability continued to decrease, showing a certain dose correlation. When the concentration of kaempferol reached 80 μm and 160 μm, cell viability decreased significantly, which was statistically significant compared with the 0 μm (vehicle) (*p*＜0.05). This indicated that when the concentration of kaempferol was 80 μm, significant cytotoxicity began to appear. Then, we observed the effect of kaempferol pre-treatment in a safe dose range on cell viability after A/R. As shown in Figure 1B, the cell viability after A/R insult was significantly decreased compared with the control group (*p*＜0.05). Different doses of kaempferol could increase the cell viability after A/R in a dose-dependent manner. Among them, the effect of improving cell viability was the best when kaempferol concentration was 40 μm. In the subsequent experiments *in vitro*, 40 μm was used as the intervention concentration of kaempferol. However, the effect of kaempferol (40 μm) on cell viability could be reversed by SIRT 1 inhibitor EX527 or PGC-1α inhibitor SR-18292 significantly as shown in Figure 1C (*p*＜0.05).

### Kaempferol Suppresses Oxidative Stress in Alveolar Epithelial Cells After A/R

**FIGURE 2:**
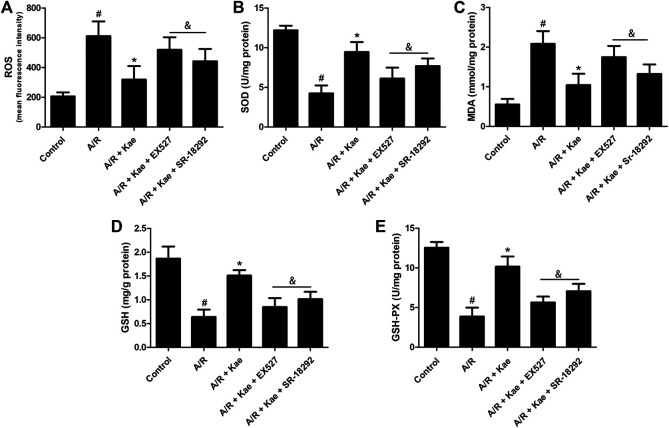
Effects of kaempferol pre-treatment on oxidative stress of L2 cells after A/R insult. **(A)** The level of ROS in L2 cells of each group. **(B)** The concentration of SOD in L2 cells of each group. **(C)** The concentration of MDA in L2 cells of each group. **(D)** The concentration of GSH in L2 cells of each group. **(E)** The concentration of GSH-PX in L2 cells of each group. Data are presented as the means ± standard error of the mean for six independent experiments. ^#^ *p*＜0.05 vs. the control group, *p＜0.05 vs. the A/R group, ^&^ *p*＜0.05 vs. the A/R + Kae group.

The degree of oxidative stress was evaluated by measuring the levels of ROS, MDA, SOD, GSH and GSH-PX in L2 cells. As shown in Supplementary Figures S1A–E, kaempferol has no obvious effects on ROS, MDA, SOD, GSH and GSH-PX in L2 cells under normal conditions (*p*＞0.05 vs. control group). As shown in Supplementary Figure S2 and Figure 2, after suffering A/R, the levels of ROS and MDA in L2 cells were significantly increased (*p*＜0.05 vs. control group), while the levels of SOD, GSH and GSH-PX were significantly decreased (*p*＜0.05 vs. control group), suggesting that the A/R process caused violent oxidative stress in L2 cells. Kaempferol pre-treatment significantly decreased ROS and MDA levels, and increased SOD, GSH and GSH-PX levels (*p*＜0.05 vs. A/R group), suggesting that kaempferol could effectively reduce oxidative stress induced by A/R. However, the effect of kaempferol on anti-oxidative stress could be significantly attenuated by EX527 or SR-18292 (*p*＜0.05 vs. A/R + Kae group).

### Kaempferol Inhibits Apoptosis of Alveolar Epithelial Cells After A/R

**FIGURE 3:**
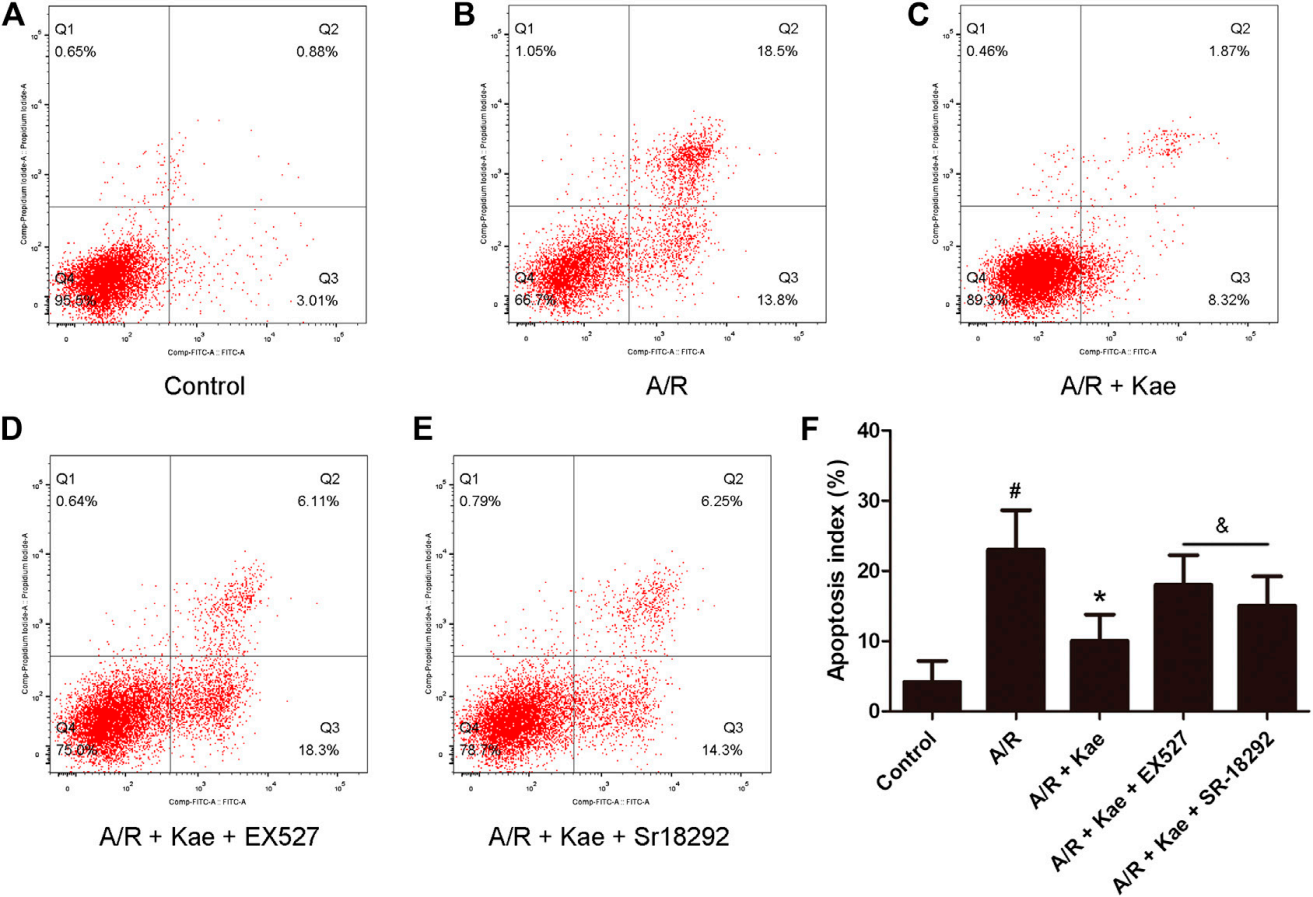
Effects of kaempferol pre-treatment on apoptosis of L2 cells after A/R insult. Flow cytometry was used to detect the apoptosis of L2 cells. **(A)** The representative image of control group. **(B)** The representative image of A/R group. **(C)** The representative image of A/R + Kae group. **(D)** The representative image of A/R + Kae + EX527 group. **(E)** The representative image of A/R + Kae + SR-18292 group. **(F)** Quantitation of apoptotic cells population. Data are presented as the means ± standard error of the mean for six independent experiments. ^#^ *p*＜0.05 vs. the control group, *p＜0.05 vs. the A/R group, ^&^ *p*＜0.05 vs. the A/R + Kae group.

Flow cytometry was used to detect the apoptosis of L2 cells after A/R. As shown in Figure 3, A/R insult caused massive apoptosis of L2 cells (*p*＜0.05 vs. control group). Kaempferol pre-treatment could significantly reduce the number of apoptosis compared with A/R group (*p*＜0.05). However, EX527 and SR-18292 both could reverse the effect of kaempferol on apoptosis of L2 cells (*p*＜0.05 vs. A/R + Kae group).

### Kaempferol Ameliorates Mitochondrial Function in Alveolar Epithelial Cells After A/R

**FIGURE 4:**
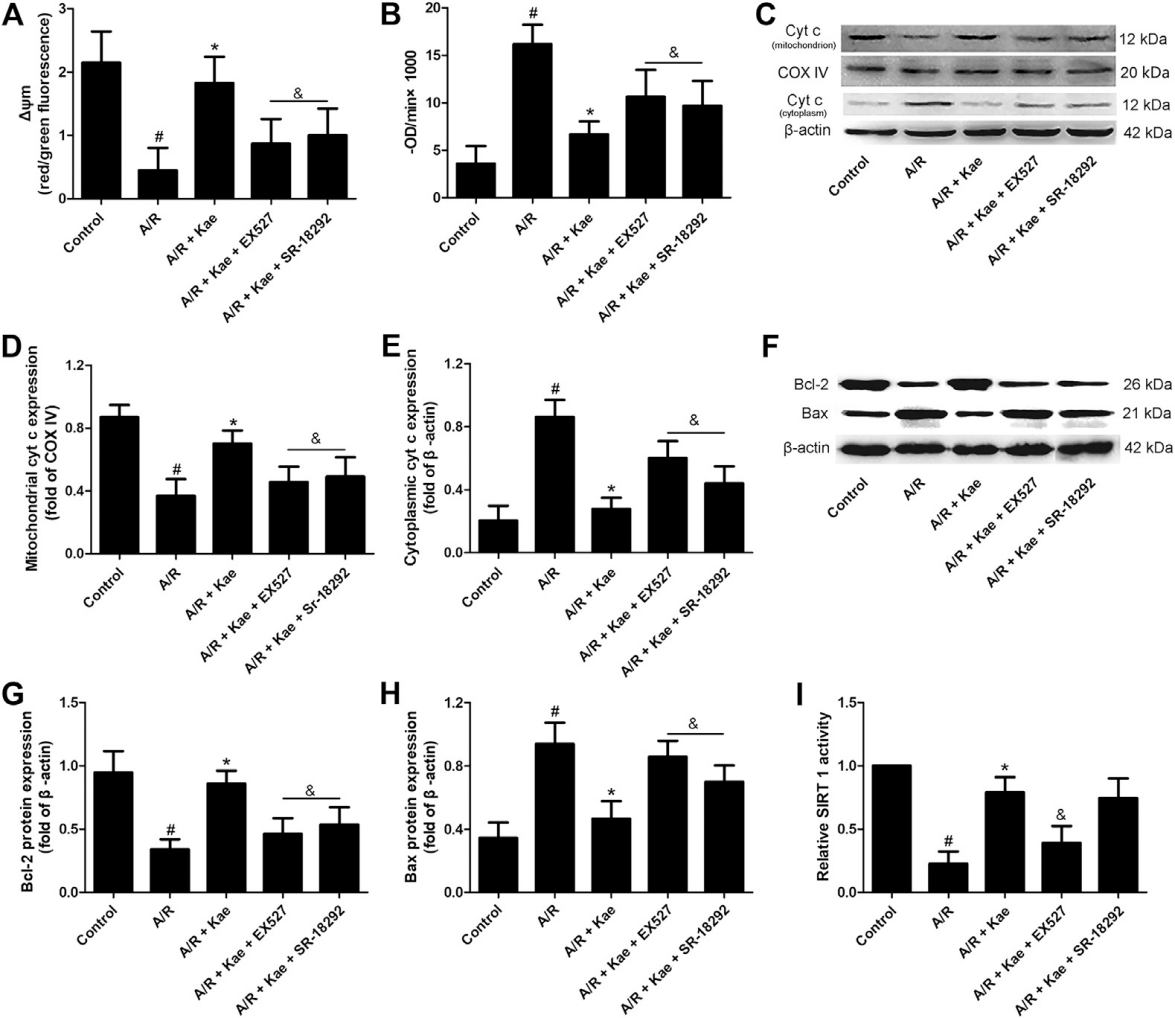
Effects of kaempferol pre-treatment on mitochondrial function of L2 cells after A/R insult. **(A)** The mitochondrial membrane potential of L2 cells in each group. The histogram of mitochondrial membrane potential depolarization which was determined by calculating the ratio of red/green fluorescence. **(B)** The mitochondrial permeability transition pores (mPTP) opening of L2 cells in each group. Changes in absorbance at 520 nm/min (ΔODmin-1) were used to express the extent of mPTP opening and analyze the intergroup differences. (ΔOD = A5,200–A52,030 min). **(C)** The representative bands of cytochrome c in mitochondria and cytoplasm of L2 cells in each group. **(D)** The relative band intensity of mitochondrial cytochrome c in each group. **(E)** The relative band intensity of cytoplasmic cytochrome c in each group. **(F)** The representative bands of Bcl-2 and Bax of L2 cells in each group. **(G)** The relative band intensity of Bcl-2 in each group. **(H)** The relative band intensity of Bax in each group. **(I)** The SIRT1 activity in each group. Data are presented as the means ± standard error of the mean for six independent experiments. ^#^ *p*＜0.05 vs. the control group, *p＜0.05 vs. the A/R group, ^&^ *p*＜0.05 vs. the A/R + Kae group.

Δψm loss and opening of mPTP are the key sign of mitochondrial dysfunction. As shown in Figures 4A,B, Supplementary Figures S1F,G, S3, S4, Kaempferol has no obvious effect on Δψm loss and opening of mPTP in L2 cells under normal conditions (*p*＞0.05 vs. control group), while A/R insult could increase the loss of Δψm and the opening of mPTP compared with the control group (*p*＜0.05). The pre-treatment of kaempferol could obviously attenuate the loss of Δψm and the opening of mPTP (*p*＜0.05 vs. A/R group). However, the increased in Δψm and the opening of mPTP were observed in EX527 or SR-18292 co-administration group (*p*＜0.05 vs. A/R + Kae group), which indicated that EX527 or SR-18292 abolished the effects of kaempferol on Δψm or mPTP.

What’s more, western blotting was used to evaluate the expressions of Bcl-2, Bax and cytochrome c in L2 cells. As shown in Figures 4C–F, A/R insult down-regulated the protein expression levels of Bcl-2 and mitochondrial cytochrome c, while up-regulating the protein expression levels of Bax and cytoplasmic cytochrome c in L2 cells (*p*＜0.05 vs. control group). Kaempferol pre-treatment could significantly up-regulate the protein expression levels of Bcl-2 and mitochondrial cytochrome c, while down-regulating the protein expression levels of Bax and cytoplasmic cytochrome c in L2 cells (*p*＜0.05 vs. A/R group). However, the regulatory effects of kaempferol on above apoptosis-related proteins can be significantly reversed by EX527 or SR-18292 (*p*＜0.05 vs. A/R + Kae group).

### Kaempferol Increases the Activity of SIRT 1 in Alveolar Epithelial Cells After A/R

As shown in Figure 4I and Supplementary Figure S1H, kaempferol could significantly increase the activity of SIRT1 in L2 cells under normal conditions (*p*＜0.05 vs. control), while A/R insult obviously decreased the activity of SIRT 1 in L2 cells compared with the control group (*p*＜0.05). Kaempferol pre-treatment could significantly increase the activity of SIRT1 in L2 cells compared with the A/R group (*p*＜0.05). As expected, the activity of SIRT 1 in group of A/R + Kae + EX527 was significantly decreased (*p*＜0.05 vs. A/R + Kae group), which suppressed by SIRT 1 inhibitor EX527. What’s more, the activity of SIRT 1 in group of A/R + Kae + SR-18292 shown no significantly change compared with the A/R + Kae group (*p* ＞0.05).

### Kaempferol Improves LIRI in Rat

**FIGURE 5:**
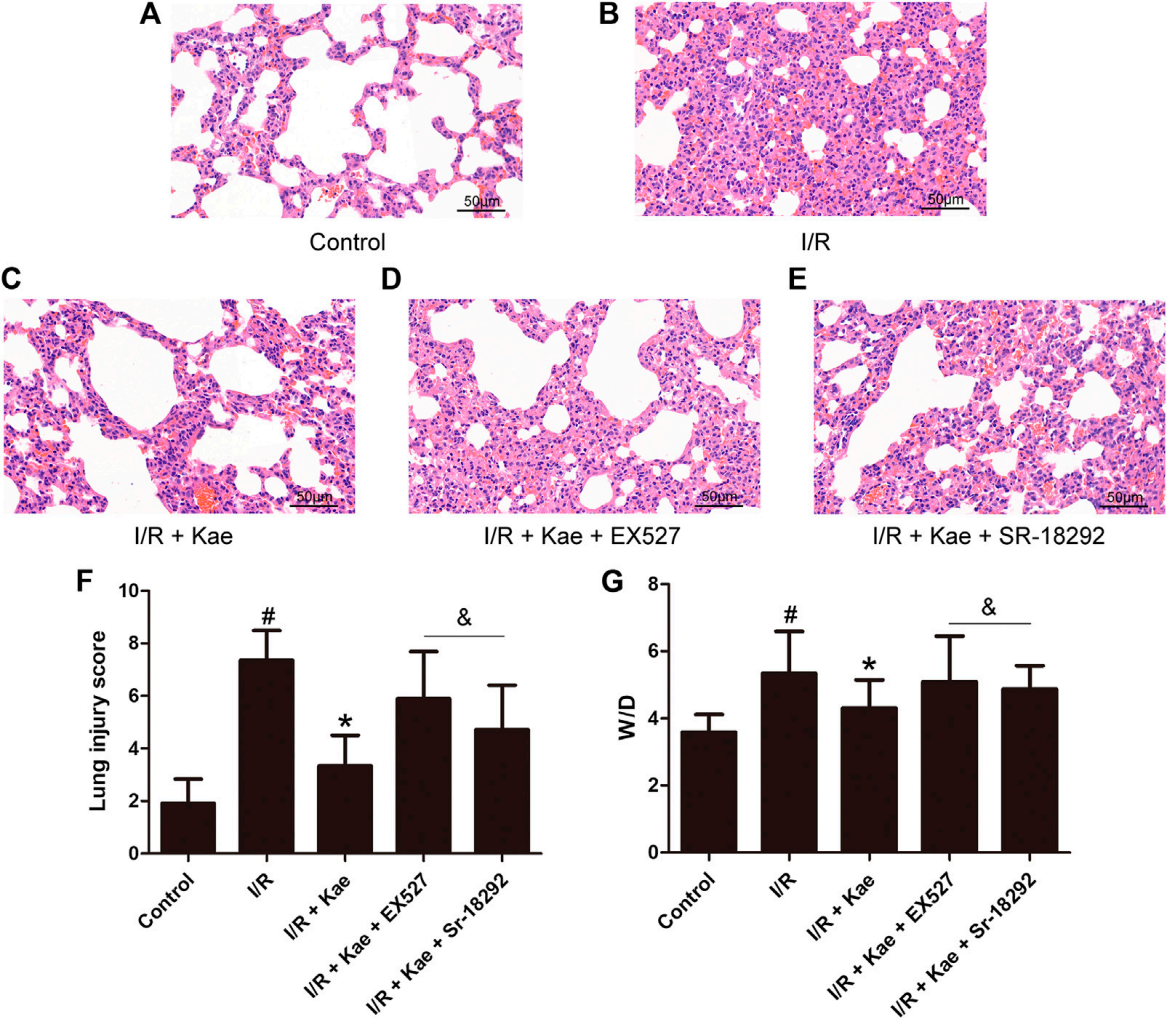
Effects of kaempferol on LIRI in rats. **(A)** The representative image of HE staining of control group (400 ×). **(B)** The representative image of HE staining of I/R group (400 ×). **(C)** The representative image of HE staining of I/R + Kae group (400 ×). **(D)** The representative image of HE staining of I/R + Kae + EX527 group (400 ×). **(E)** The representative image of HE staining of I/R + Kae + SR-18292 group (400 ×). **(F)** The lung injury score of each group. **(G)** The ratio of wet/dry of lung in each group. Data are presented as the means ± standard error of the mean for six independent experiments. *p＜0.05 vs. the I/R group, ^&^ *p*＜0.05 vs. the I/R + Kae group.

HE staining was used to evaluate the pathological changes of lung in rats. As shown in Figures 5A–F, I/R insult caused severe injury of lung tissue including alveolar structure deformation, epithelial cells degeneration, necrotic and shedding, and interstitial edema. Inflammatory cells infiltrated, a large amount of fluid exudation and more red blood cells were seen in alveoli. The lung injury score of I/R group was significantly higher than that of the sham group (*p*＜0.05). Kaempferol pre-treatment could significantly improve the lung injury of rats after I/R, and the lung injury score was significantly lower than that of I/R group (*p*＜0.05). However, both EX527 or SR-18292 could significantly reverse the effect of kaempferol on improving LIRI in rat (*p*＜0.05).

In addition, the W/D of lung was also measured. As shown in Figure 5G, the W/D ratio of I/R group was significantly increased compared with thecontrol group (*p*＜0.05), while kaempferol pre-treatment could significantly decrease the W/D ratio compared with the I/R group (*p*＜0.05). However, both EX527 or Sr18292 could significantly reverse the effect of kaempferol on the W/D ratio (*p*＜0.05).

### Kaempferol Suppresses Oxidative Stress in Lung Tissue of Rat After I/R

**FIGURE 6:**
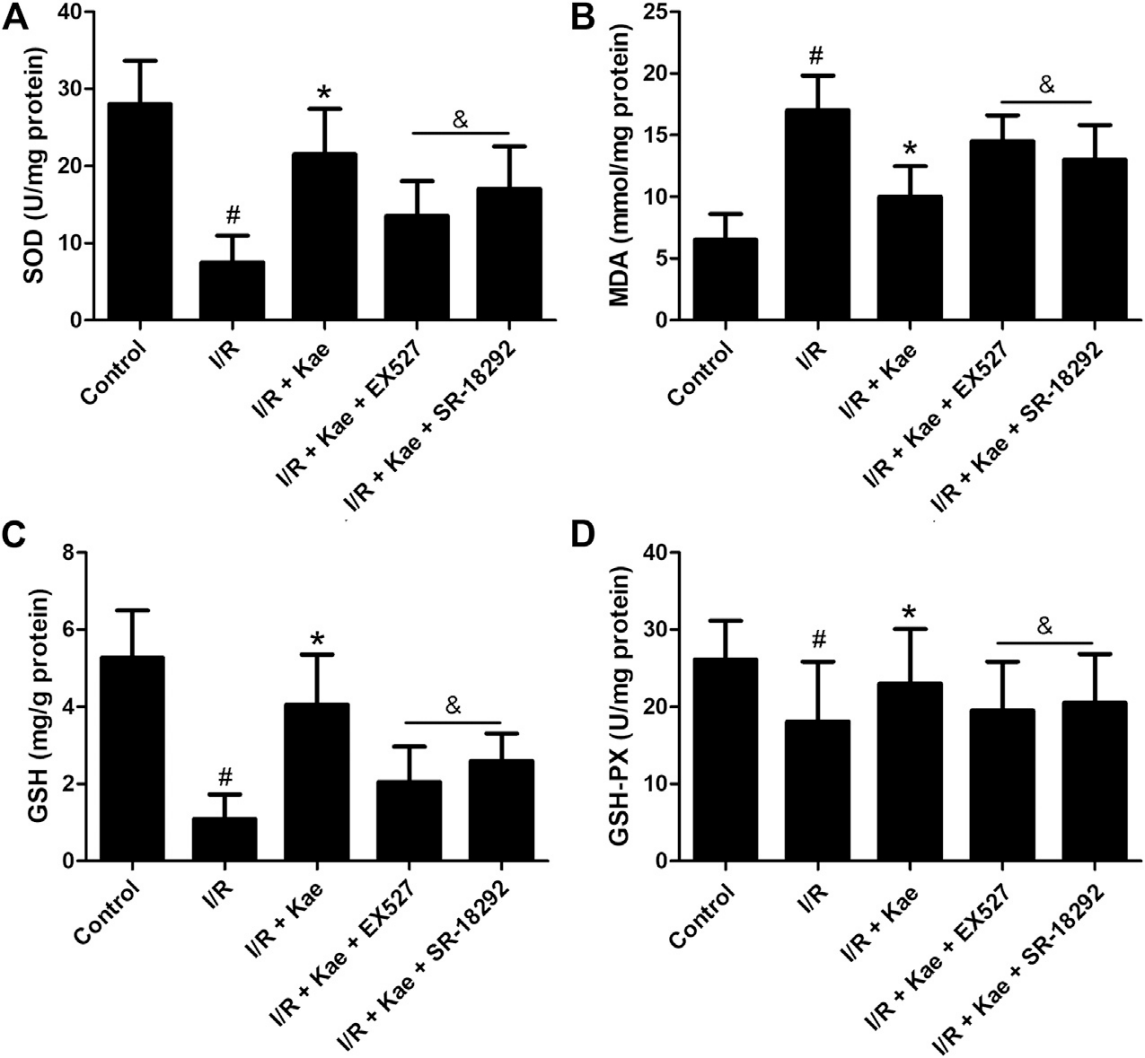
Effects of kaempferol on oxidative stress in lung tissue after I/R. **(A)** The concentration of SOD in lung of each group. **(B)** The concentration of MDA in lung of each group. **(C)** The concentration of GSH in lung of each group. **(D)** The concentration of GSH-PX in lung of each group. Data are presented as the means ± standard error of the mean for six independent experiments. *p＜0.05 vs. the I/R group, ^&^ *p*＜0.05 vs. the I/R + Kae group.

The degree of oxidative stress was evaluated by measuring the levels of MDA, SOD, GSH and GSH-PX in lung tissue of rat after I/R. As shown in Supplementary Figures S5A–D, kaempferol has no obvious effects on MDA, SOD, GSH and GSH-PX in lung tissue of normal rat. As shown in Figure 6, after suffering I/R, the level of MDA in lung tissue of rat was significantly increased, while the levels of SOD, GSH and GSH-PX were significantly decreased (*p*＜0.05 vs. the control group). Kaempferol pre-treatment significantly decreased MDA level, and increased SOD, GSH and GSH-PX levels (*p*＜0.05 vs. the I/R group), suggesting that kaempferol could effectively reduce oxidative stress induced by I/R. However, the effects of kaempferol on anti-oxidative stress in lung tissue of rat were significantly attenuated by EX527 or SR-18292 (*p*＜0.05).

### Kaempferol Inhibits Apoptosis of Lung Cells of Rat After I/R

**FIGURE 7:**
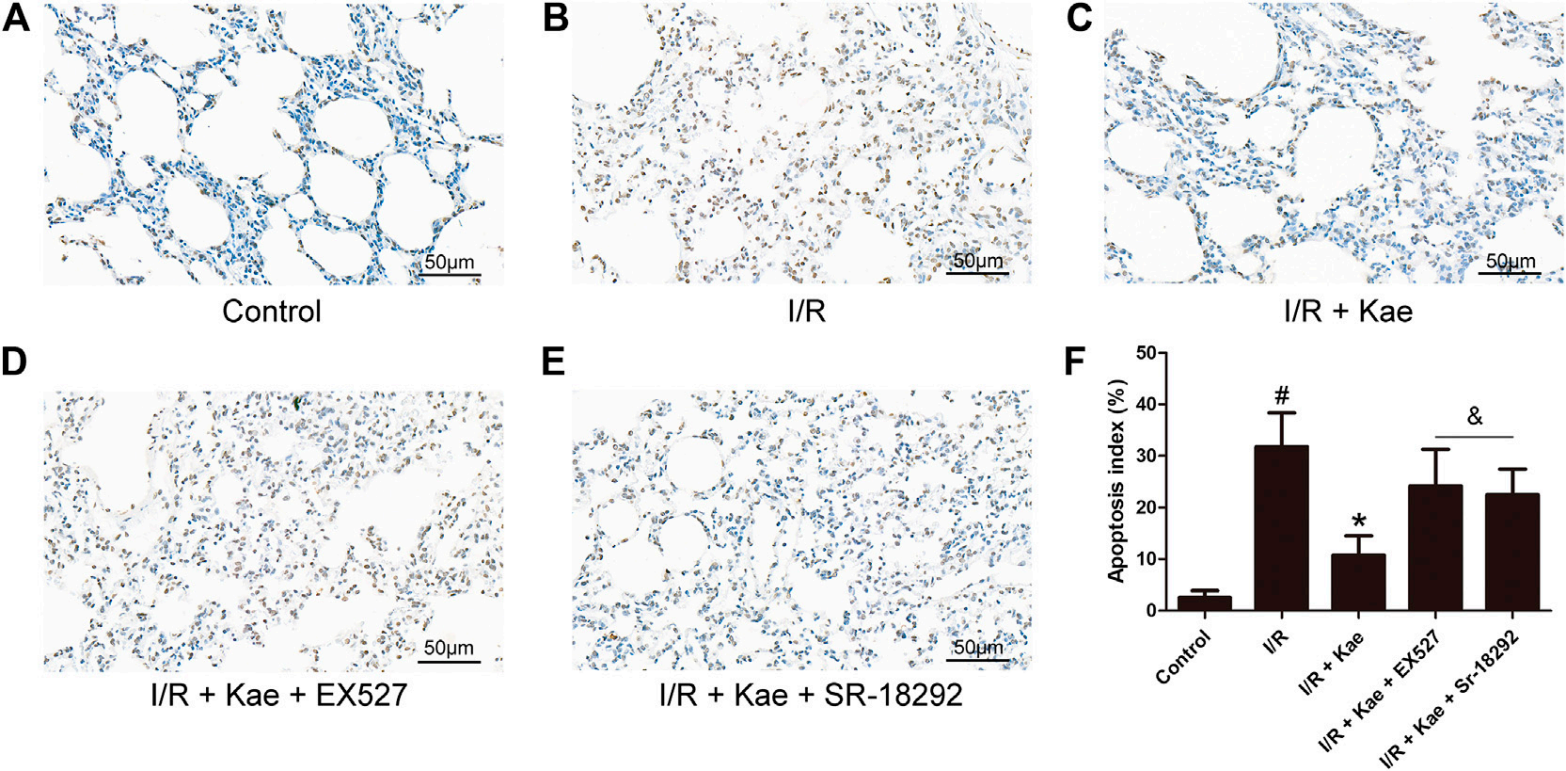
Effects of kaempferol on apoptosis in lung tissue after I/R. **(A)** The representative image of TUNEL staining of control group (400 ×). **(B)** The representative image of TUNEL staining of I/R group (400 ×). **(C)** The representative image of TUNEL staining of I/R + Kae group (400 ×). **(D)** The representative image of TUNEL staining of I/R + Kae + EX527 group (400 ×). **(E)** The representative image of TUNEL staining of I/R + Kae + SR-18292 group (400 ×). **(F)** The apoptosis index of each group. Data are presented as the means ± standard error of the mean for six independent experiments. *p＜0.05 vs. the I/R group, ^&^ *p*＜0.05 vs. the I/R + Kae group.

TUNEL staining was used to detect the apoptosis of lung cells of rat after I/R. As shown in Figure 7, I/R insult caused massive apoptosis of lung cells of rat (*p*＜0.05 vs. the control group). Kaempferol pre-treatment could significantly reduce the number of apoptosis compared with the I/R group (*p*＜0.05). However, EX527 and SR-18292 both could reverse the effect of kaempferol on apoptosis of lung cells of rat after I/R (*p*＜0.05 vs. A/R + Kae group).

### Kaempferol Regulates the Expressions of Apoptosis Related Proteins in Lung Tissue of Rat After I/R

**FIGURE 8:**
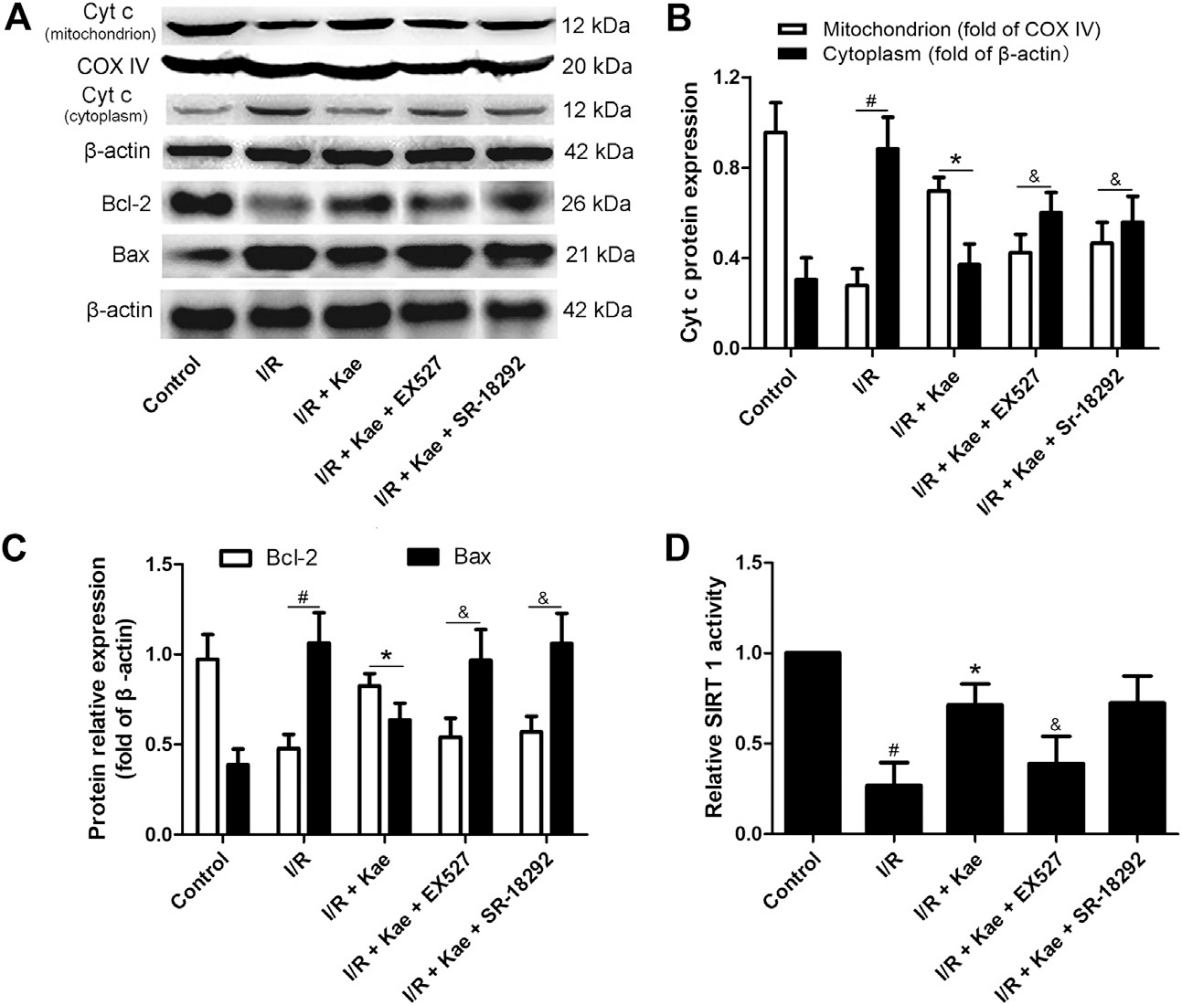
Effects of kaempferol on the protein expression levels of cytochrome c, Bcl-2 and Bax in lung after I/R. **(A)** The representative bands of cytochrome c, Bcl-2 and Bax. **(B)** The relative band intensity of cytochrome c in mitochondria and cytoplasm in each group. **(C)** The relative band intensity of Bcl-2 and Bax in each group. **(D)** The SIRT1 activity in each group. Data are presented as the means ± standard error of the mean for six independent experiments. *p＜0.05 vs. the I/R group, ^&^ *p*＜0.05 vs. the I/R + Kae group.

Western blotting was used to evaluate the expressions of Bcl-2, Bax and cytochrome c in lung tissue of rat after I/R. As shown in Figures 8A–C, I/R insult down-regulated the protein expression levels of Bcl-2 and mitochondrial cytochrome c, while up-regulating the protein expression levels of Bax and cytoplasmic cytochrome c in lung tissue (*p*＜0.05 vs. the sham group). Kaempferol pre-treatment could significantly up-regulate the protein expression levels of Bcl-2 and mitochondrial cytochrome c, while down-regulating the protein expression levels of Bax and cytoplasmic cytochrome c in lung tissue (*p* ＜0.05 vs. I/R group). However, the regulatory effects of kaempferol on apoptosis-related proteins can be significantly reversed by EX527 or SR-18292 (*p*＜0.05 vs. I/R + Kae group).

### Kaempferol Increases the Activity of SIRT 1 in Lung Tissue of Rat After I/R

As shown in Supplementary Figure S5E and Figure 8D, kaempferol could significantly increase the activity of SIRT 1 in lung tissue of normal rats (*p*＜0.05 vs. control), while I/R insult obviously decreased it after I/R compared with the control group (*p*＜0.05). Kaempferol pre-treatment could significantly increase the activity of SIRT1 in lung tissue of rat compared with the I/R group (*p*＜0.05). As expected, the activity of SIRT 1 in group of I/R + Kae + EX527 was significantly decreased (*p*＜0.05 vs. I/R + Kae group), which was suppressed by SIRT 1 inhibitor EX527. What’s more, the activity of SIRT 1 in group of I/R + Kae + SR-18292 shown no significantly change compared with the I/R + Kae group (*p*＜0.05).

## Discussion

It is known that oxidative stress is involved in the process of I/R injury (Dröge, 2002; Ferrari and Andrade, 2015). Studies have shown that energy consumption and accumulation of toxic oxygen metabolites are the main causes of tissue injury during ischemia (Zimmerman and Granger, 1994). During tissue ischemia, xanthine dehydrogenase is converted to xanthine oxidase, and xanthine oxidase relies on oxygen to metabolize hypoxanthine. When hypoxanthine is supplied by reperfusion (reoxygenation), ROS will be formed (Zimmerman and Granger, 1994). In addition, hydrogen peroxide produces highly toxic hydroxyl radicals through Haber-Weiss reaction, which will be aggravated by the increase of free iron during ischemia (Iadecola and Anrather, 2011). ROS and other free radicals will cause serious damage to tissue. Unlike the other organs, the alveoli in the lung can maintain erobic metabolism and prevent hypoxia. Therefore, the oxidative stress caused by lung ischemia is different from the oxidative stress caused by hypoxia (de Perrot et al., 2003). During ischemia, mitochondrial function is inhibited, ATP in lung cells is decreased, and its degradation products (hypoxanthine, etc.) are increased, and excessive ROS will be produced during reperfusion. The increase of ROS is an important characteristic of LIRI, which makes a key contribution to pulmonary failure (Sun et al., 2011). Importing anti-oxidants to balance the production of ROS is an important strategy to reduce the toxicity caused by excessive ROS (McCord and Fridovich, 1969). As a main anti-oxidant in the body, SOD can catalyze the conversion of superoxide to oxygen and hydrogen peroxide, which will be converted into water and oxygen by other anti-oxidant proteins including thioredoxin, catalase and glutathione peroxidase (Wang et al., 2018). In this study, ROS and MDA levels increased, SOD, GSH and GSH-PX levels decreased in L2 cells after A/R, suggesting that A/R triggered severe oxidative stress in alveolar epithelial cells. The oxidative stress reaction in L2 cells pre-treated with kaempferol was effectively suppressed, and the release of ROS were significantly reduced. *In vivo*, the level of oxidative stress in the lungs of rats after I/R was also significantly suppressed by kaempferol. These results suggest that kaempferol can effectively eliminate the excessive production of ROS induced by I/R, inhibit oxidative stress reaction, and protect lung cells from I/R insult.

Studies have shown that apoptosis is closely related to the process of LIRI (Martin et al., 2003; Rivo et al., 2007). Apoptosis is a form of cell death, which is determined by genes and can also be triggered by external factors. Different species have different mechanisms for regulating cell death, but they are all regulated by the homologous proteins and involve mitochondria (Estaquier et al., 2012). Unlike cell necrosis, apoptosis does not occur during ischemia, but reaches a peak during reperfusion (Stammberger et al., 2000). The endogenous pathway of apoptosis depends on mitochondria and is activated by excessive ROS and oxidative stress (Galluzzi et al., 2018). In addition, cell apoptosis will generate a feedback loop that interacts with oxidative stress, leading to continuous expansion of tissue damage (Tuder et al., 2003). Bcl-2 family members are key regulators of mitochondrial apoptosis pathway, which are considered to play a key role in oxidative stress-induced apoptosis (Wong and Puthalakath, 2010). Bcl-2 exerts anti-apoptotic effects by counteracting pro-apoptotic proteins produced by Bax digging holes in the outer membrane of mitochondria, inhibiting the release of cytochrome c to the cytoplasm, and suppressing the cytochrome c - mediated caspase cascade (Kontos et al., 2014). Bcl-2 also directly participates in energy metabolism by regulating respiratory chain and terminal voltage dependent anion channels through cyclooxygenase (Elsässer et al., 2004). In this study, a large number of apoptosis occurred in L2 cells after A/R injury. The expressions of Bcl-2 and mitochondrial cytochrome c decreased, while the expressions of Bax and cytoplasmic cytochrome c increased in L2 cells. Kaempferol pre-treatment could inhibit the number of apoptosis and reverse the protein expression levels of Bcl-2, Bax and cytochrome c in mitochondria or cytoplasm. In the *in vivo* experiment of the rat I/R model, we have obtained consistent results with *in vitro*. It is suggested that apoptosis via mitochondria-dependent pathway is involved in the LIRI process, and kaempferol can inhibit I/R-induced apoptosis of lung cells through mitochondria-dependent pathway.

Mitochondrial dysfunction and damage are associated with a variety of human diseases including acute lung injury (Wang et al., 2015). Mitochondria are involved in the integration and circulation of intracellular death signals, including oxidative stress and apoptosis (Liu and Chen, 2017). Mitochondria are the main target of I/R injury. After reperfusion occurs, ROS accumulates excessively, causing oxidative stress. This oxidative stress induces increase of Ca^2+^, leading to increased mitochondrial inner membrane permeability and loss of mitochondrial membrane potential. Mitochondria swell and release cytochrome c and apoptotic protease activators into the cytoplasm, which together activate caspases and induce apoptosis (Murphy et al., 1999; Solaini et al., 2010). Therefore, mitochondria are potential target organelles for the treatment of LIRI. PGC-1α is one of the key regulators of mitochondrial function. It is a co-factor of PPAR family transcription factors, which can increase the expression of mitochondrial fatty acid oxidation genes (Finck and Kelly, 2007). It also binds and activates nuclear respiratory factor 1 (NRF1), a transcription factor that increases oxidative phosphorylation, and stimulates the synthesis of transcription factor A mitochondrial (TFAM), which leads to mitochondrial DNA transcription, maintenance and replication, and regulates mitochondrial biogenesis (Scarpulla, 2008). The study also found that PGC-1α contributes to energy production and prevents ROS insult (Jian et al., 2011). Sirtuins are a class of NAD + -dependent histone deacetylases, which are expressed in all eukaryotic cells and participate in the regulation of cellular stress response, mitochondrial biogenesis, apoptosis and inflammation (Maksin-Matveev et al., 2014). SIRT 1 is the mammalian ortholog of yeast SIR 2, which is mainly concentrated in the nucleus, and its main responsibility is to deacetylate histones (Rahman and Islam, 2011). SIRT 1 can catalyze the deacetylation of PGC-1α, thereby affecting cell metabolism and mitochondrial biogenesis (Nemoto et al., 2005). The expression of SIRT 1 was decreased during I/R injury, and the injury caused by I/R could be alleviated by up-regulating SIRT1 (Assadiasl et al., 2020). SIRT1 has become a potential target to prevent I/R injury.

In previous studies, we have confirmed that kaempferol exerts anti-LIRI by increasing SIRT1 activity (Yang et al., 2020). In this study, we found that kaempferol can improve the stability of mitochondrial membrane potential and inhibit the opening of mPTP of L2 cells after A/R, up-regulate the levels of Bcl-2 and mitochondrial cytochrome c while down-regulating the levels of Bax and cytoplasmic cytochrome c in L2 cells after A/R or lung tissue of rats after I/R. After the SIRT 1 inhibitor EX527 was used to inhibit the activity of SIRT 1 protein, the above effects of kaempferol were attenuated. After the PGC-1α inhibitor SR-18292 was used to inhibit PGC-1α deacetylation, the effects of kaempferol on improving mitochondrial function, anti-oxidative stress and anti-apoptosis were also reversed. However, the protein activity of SIRT1 was not affected. All above results indicate that in the process of LIRI, kaempferol pre-treatment can target to increase the activity of SIRT 1 protein, thereby regulating PGC-1α, improving mitochondrial function, inhibiting the release of ROS, resisting oxidative stress, inhibiting apoptosis, and finally exerting anti-LIRI effect.

In conclusion, in this study, we confirmed that kaempferol can improve mitochondrial dysfunction during LIRI, inhibit ROS over-production, reduce oxidative stress and inhibit lung cell apoptosis *in vivo* and *in vitro*. However, both SIRT 1 inhibitor EX527 and PGC-1α inhibitor SR-18292 could reverse the above effects of kaempferol, and SR-18292 did not affect the effect of kaempferol on SIRT1 protein activity. These results indicate that the effect of kaempferol anti-LIRI involves the SIRT 1/PGC-1α/mitochondria signaling pathway.

## Data Availability

The original contributions presented in the study are included in the article/Supplementary Material, further inquiries can be directed to the corresponding author.
