# Supplementary figures and images for: Kaempferol Alleviates Oxidative Stress and Apoptosis Through Mitochondria-dependent Pathway During Lung Ischemia-Reperfusion Injury
Source: Front Pharmacol. 2021 Mar 4;12:624402. doi: 10.3389/fphar.2021.624402 (PMC7969663; doi:10.3389/fphar.2021.624402)

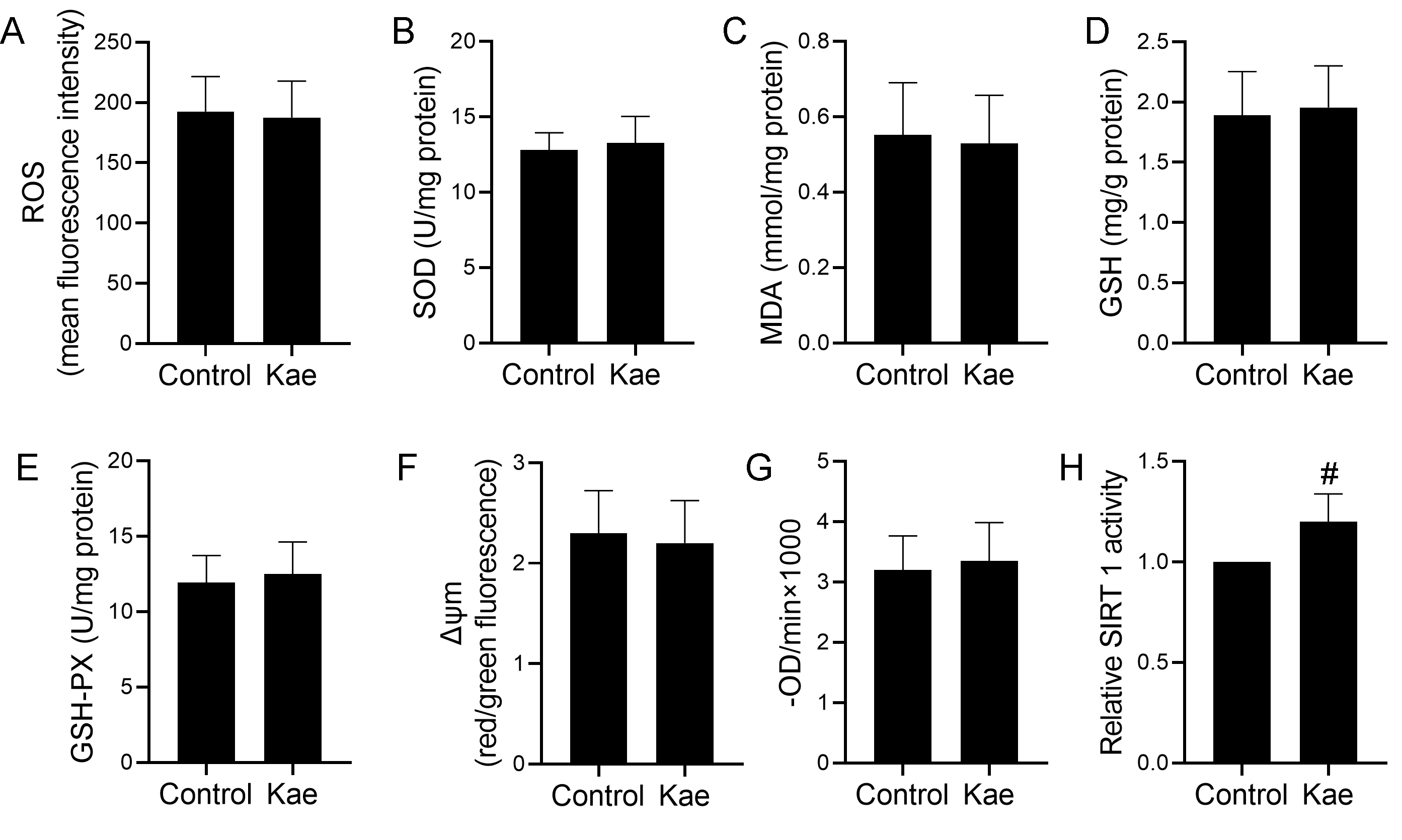

Supplement: Supplementary file 1 [file image1.tif]

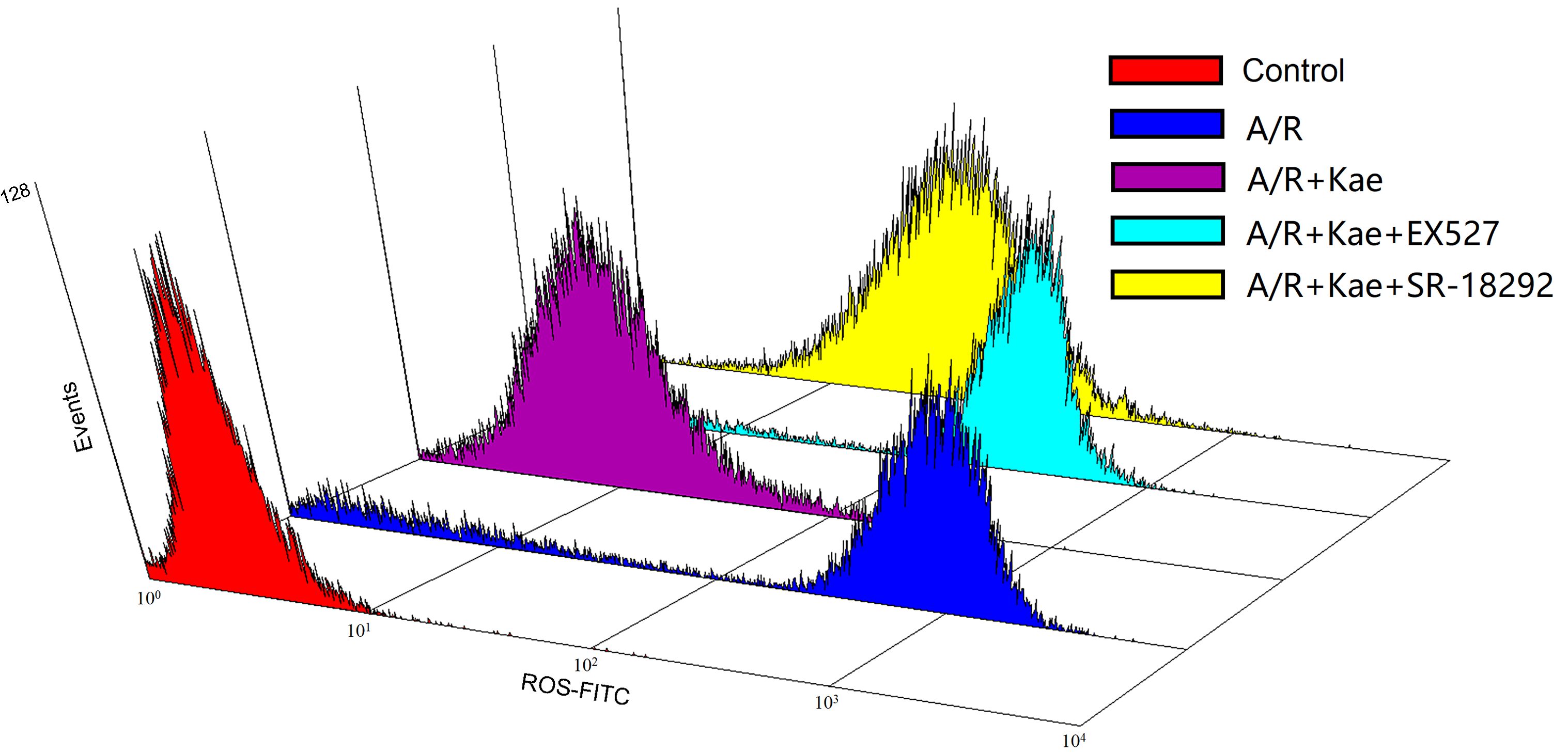

Supplement: Supplementary file 2 [file image2.tif]

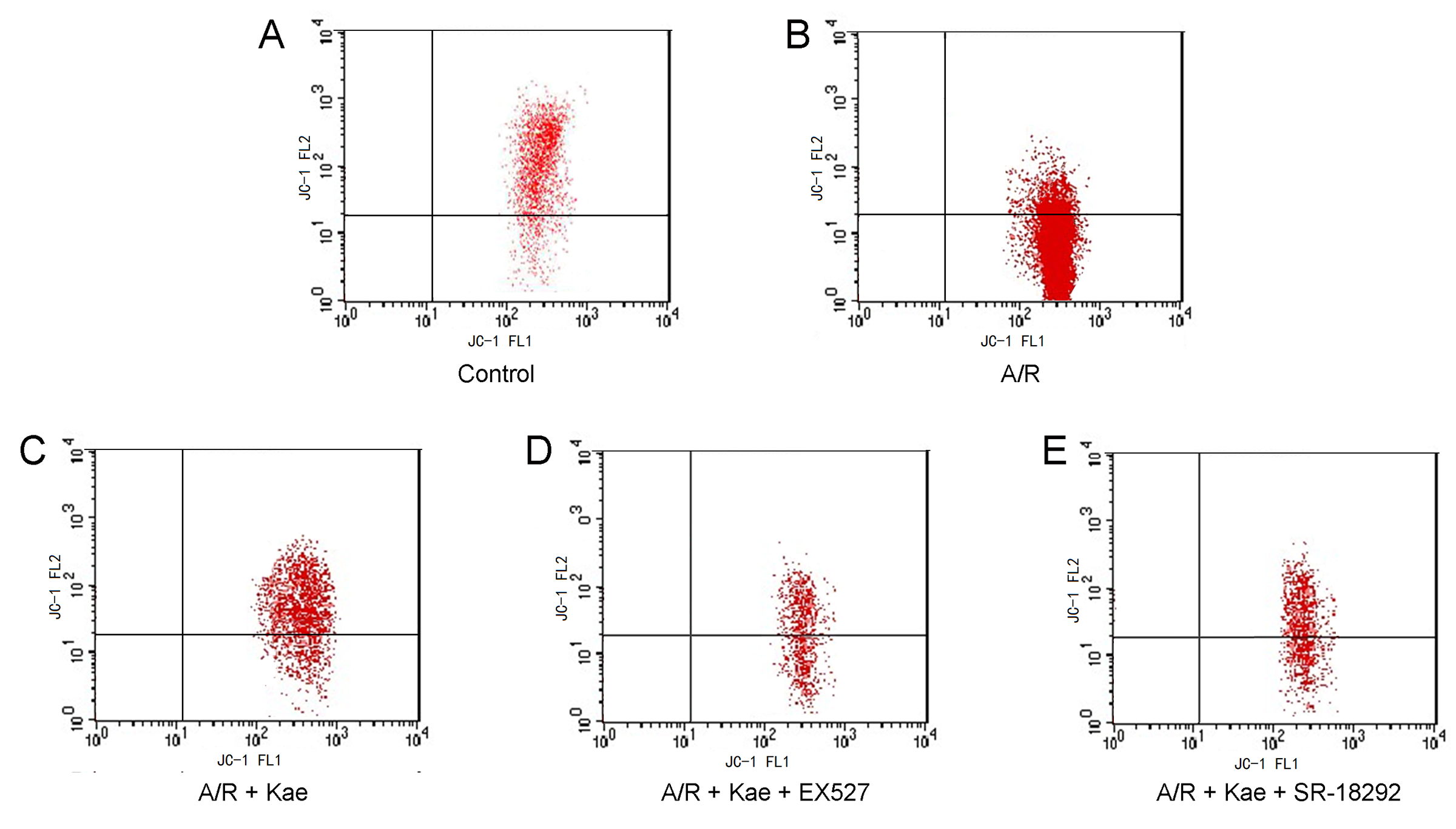

Supplement: Supplementary file 3 [file image3.tif]

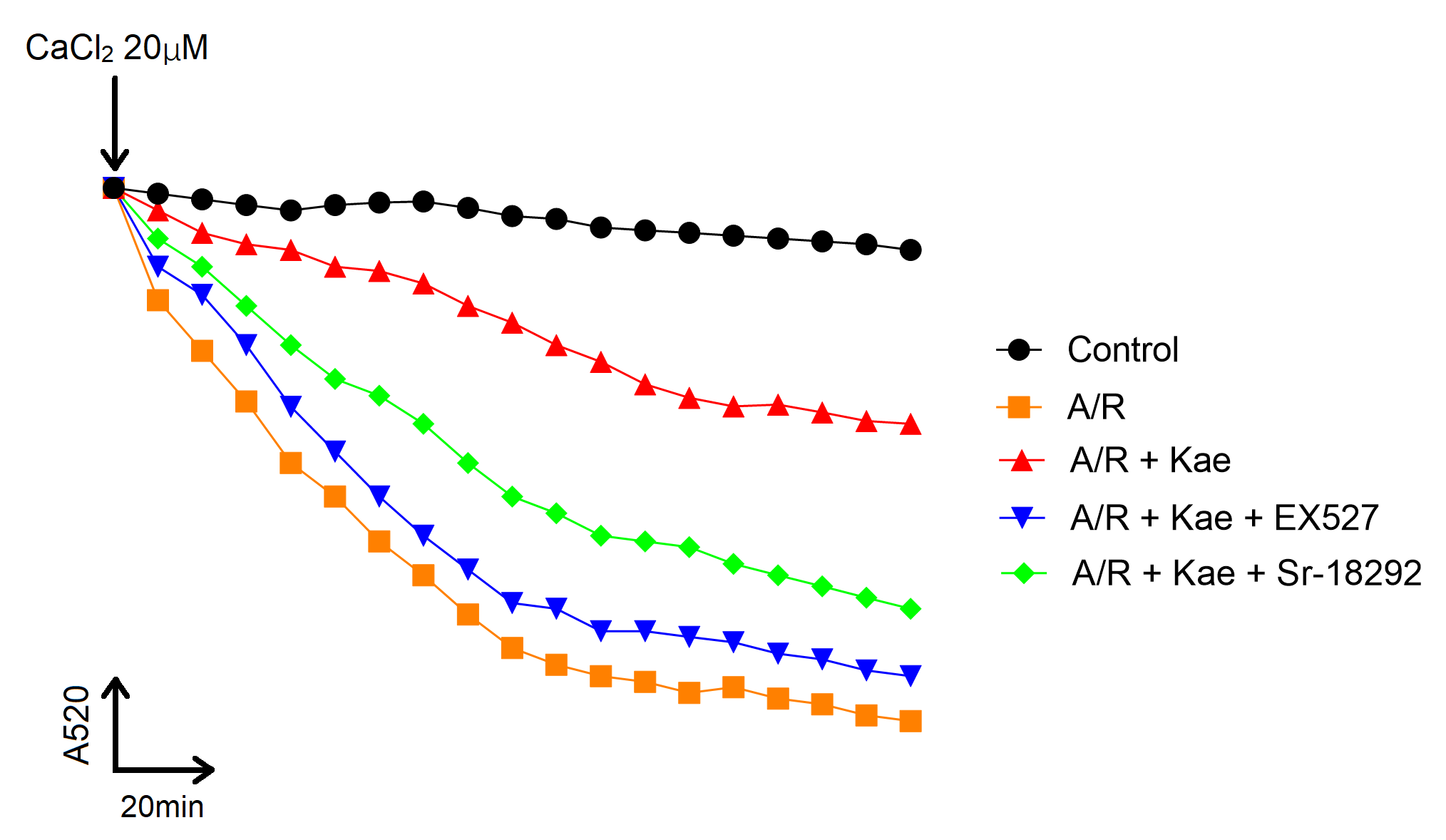

Supplement: Supplementary file 4 [file image4.tif]

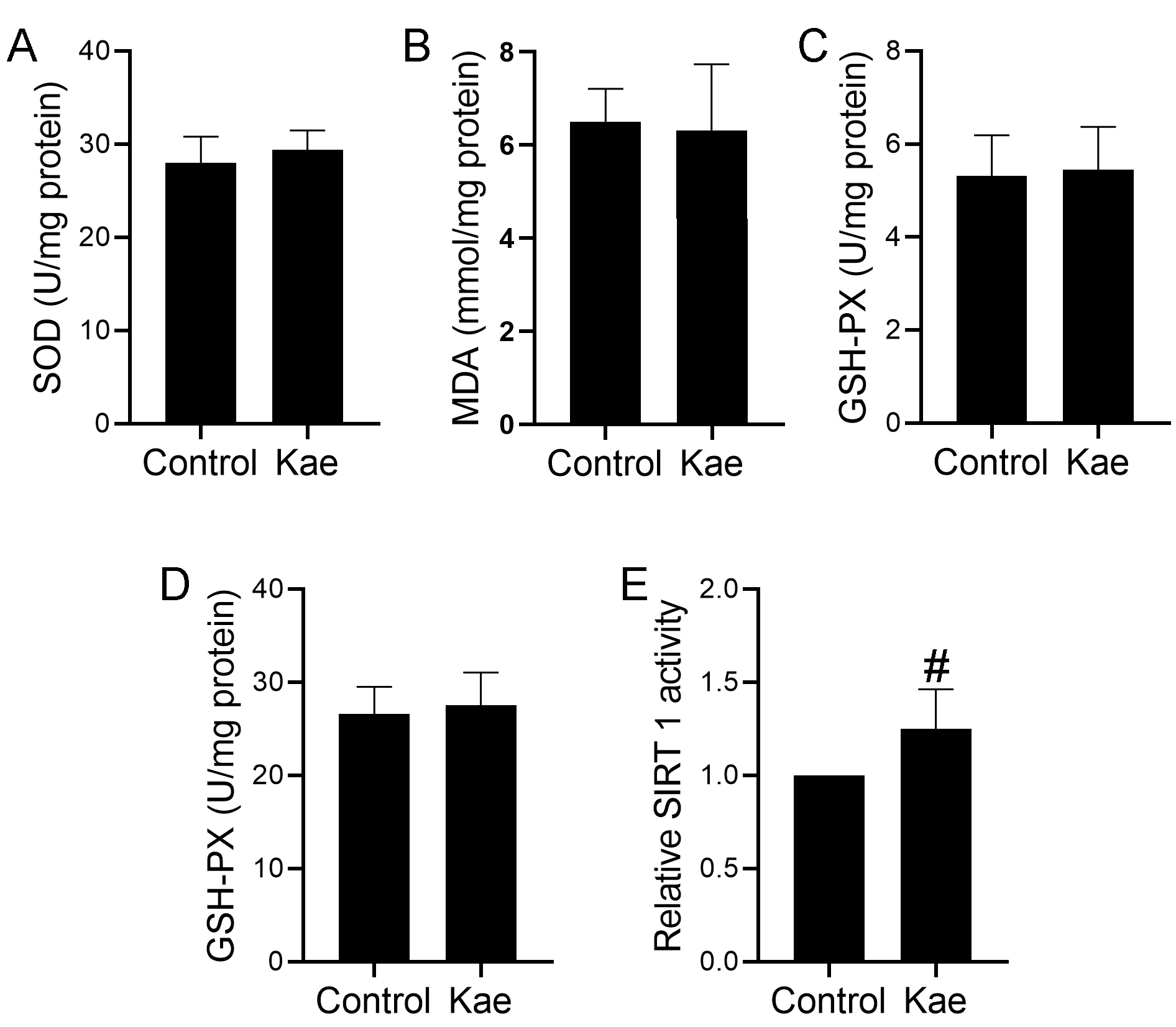

Supplement: Supplementary file 5 [file image5.tif]
